# Harnessing marine microbiomes to develop a sustainable, all‐Atlantic bioeconomy

**DOI:** 10.1002/mlf2.12124

**Published:** 2024-05-27

**Authors:** Cristiane Thompson, Alice C. Ortmann, Henk Bolhuis, Thulani Makhalanyane, Fabiano Thompson

**Affiliations:** ^1^ Institute of Biology Federal University of Rio de Janeiro (UFRJ) Rio de Janeiro Brazil; ^2^ Fisheries and Oceans Canada Bedford Institute of Oceanography Dartmouth Nova Scotia Canada; ^3^ Department of Marine Microbiology and Biogeochemistry Royal Netherlands Institute for Sea Research (NIOZ) The Netherlands; ^4^ Department of Microbiology, School of Data Science and Computational Thinking Stellenbosch University Stellenbosch South Africa

Marine microbiomes are integral to the functioning of a healthy ocean and have the potential to be key contributors toward an all‐Atlantic sustainable blue bioeconomy (Figure 1). New compounds may be developed based on these microbiomes. The Atlantic Ocean is a biodiversity hotspot. The All‐Atlantic Ocean Research and Innovation Alliance (AAORIA) Declaration, signed in 2022 in Washington, DC, is a promising mechanism to move forward marine microbiome science, technology, and innovation.

**Figure 1:**
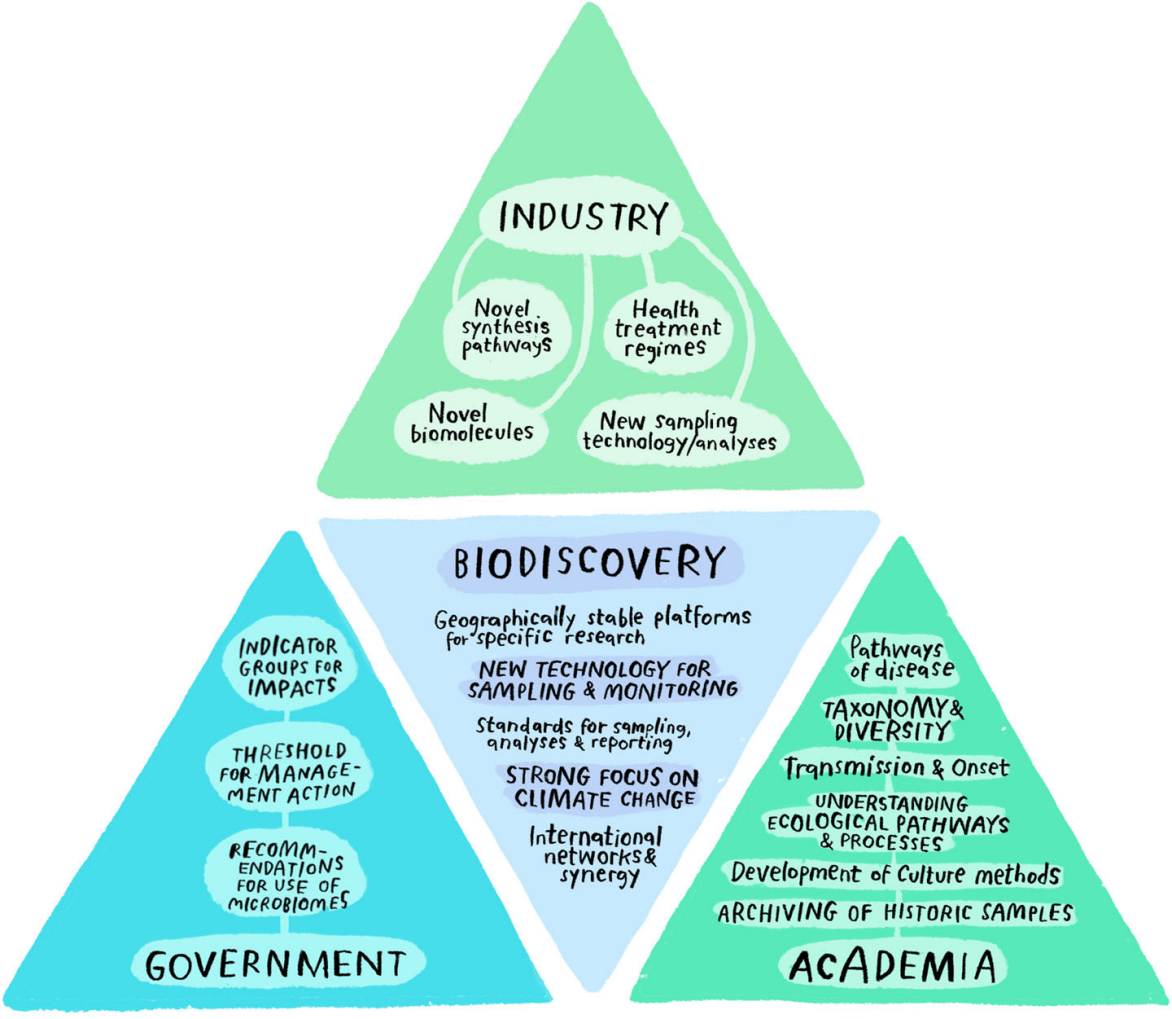
Harnessing marine microbiomes and the triple helix (academia+government+industry). The citizens are represented in each helix. Citizens may be engaged by means of ocean literacy and communication actions. Artwork by Rán Flygenring.

However, science, technology, and innovation initiatives in the South Atlantic require great attention. Here we highlight the potential, and challenges ahead, of an all‐Atlantic marine microbiome initiative. Successful examples around the world are discussed, and suggestions are put forward.

Microbes including bacteria, archaea, protists, and fungi represent the majority of the ocean biomass^1^. These microbes play a central a role in nutrient cycling, fluxes of energy, and matter in the ocean contributing to primary production and climate change mitigation^2^. Furthermore, long‐term host–microbe co‐evolution has allowed (in)vertebrates to occupy diverse niches in the ocean (e.g., the deep sea, hydrothermal vents, and coral reefs), facilitated by endosymbiotic microbes which have established stable partnerships with their hosts^3^. The Tara Ocean global survey demonstrated that microbial diversity is modulated by water mass parameters, such as temperature and nutrients^4^. More recently, the Tara Research Federation has identified a wealth of novel pico‐planktonic eukaryotic primary producers inhabiting oxygen minimum zones^5^. Simultaneously, databases and artificial intelligence tools have been developed for mining marine microbiomes^6^. Some of these developments are being carried out in collaboration with the industry to expand knowledge of marine microbiomes. While there is much more to discover about marine microbiomes, we know they are important players in supporting marine food webs. We also know that their functional diversity is remarkably high and that these microbiomes are integral for a healthy ocean which may support a sustainable bioeconomy. In this context, we believe that countries bordering the Atlantic Ocean (including representatives from the Global South and North) may create meaningful partnerships to provide the investment and science innovation needed to address several of the United Nations Sustainable Development Goals.

## THE RATIONALE FOR HARNESSING MARINE MICROBIOMES FOR ENVIRONMENTAL HEALTH, FOOD, AND DRUG PRODUCTION

Almost 900 million people are affected by hunger (http://wfp.org; accessed April 10, 2024). Further pressure on traditional agriculture and aquaculture practices is foreseen due to human population increase, which is estimated to approach up to 10 billion people in 2050. While aquaculture is responsible for the annual production of 88 million metric tonnes of fish, most are produced in inland (fresh) waters. Estimates suggest that <40% of the fish production is derived from marine systems (mariculture). Harvesting wild populations of marine organisms cannot sustainably provide sufficient additional food. Estimates suggest that 35.4% of stocks may be overfished as of 2019^7^. Alternative approaches to mariculture and coastal agriculture are urgently required. These approaches may harness the diverse metabolic traits of microbial communities to augment the production of marine‐derived resources^8^.

Saline agriculture may provide a sustainable alternative to traditional coastal agriculture (https://Simbaproject.eu; https://www.thesaltdoctors.com/). These approaches contribute to sustainability by reducing the need for freshwater and by making use of less arable land. To promote saline agriculture, different initiatives have applied high‐tech microbiological screening for plant growth‐promoting genes from salt‐tolerant microbes. These microbes may alter the root habitats or modify the plant physiology, increasing production in salt‐impacted land. Microbiomes are also central for the development of low‐carbon and integrated multitrophic mariculture (IMTA). These technologies focus on the developing multitrophic systems that recycle waste and reduce antibiotic use and carbon emissions^8^. Biofloc technology (BFT) has also been successfully applied in the shrimp aquaculture^9^. BFT is based on the use of microbial consortia which can serve as a food source and to remediate rearing water quality.

Marine microbiomes also offer great potential for drug discovery^10^. Antibiotic resistance, the emergence of new diseases, and increasing cancer rates require the development of novel pharmaceuticals to address these public health challenges. Currently, there are >18,000 natural products which have been derived from the marine environments (microorganisms and macroorganisms)^11^. Of these, over 5000 products have been patented^11^. Estimates suggest that over 40,000 novel gene clusters, obtained from 35,000 microorganisms and macroorganisms, may code for highly novel bioactive molecules and enzymes. On this path, the Blue Remediomics Initiative aims to develop tools to catalog marine microbiome datasets, new medicines, cosmeceuticals, and bioproducts (https://www.embl.org/news/science/blueremediomics-harnessing-marine-microbes-to-promote-a-circular-bioeconomy/). However, some marine microbiomes remain poorly characterized. A recent initiative has put forward standard methods and best practices for marine microbiome science, technology, and innovation (STI) (https://www.frontiersin.org/research-topics/15877/marine-microbiomes-towards-standard-methods-and-best-practices). Research priorities in marine microbiomes include measuring microbiome diversity in poorly sampled regions including the deep sea and Global South, quantifying interactions between microbiota, and harnessing oceanic microorganisms to expand the bioeconomy. These insights are crucial for efforts aimed at driving policy and increasing ocean literacy^4^.

## BENEFITS AND LIMITATIONS OF THE EXISTING APPROACHES

A series of international agreements have been signed to support collaborative science in the Atlantic^12^ (Figure 1). A consensus outcome of the All‐Atlantic Declaration is the establishment of the AAORIA to support and facilitate efforts aimed at the sustainable development of the Atlantic Ocean. The recent announcement that AAORIA will focus on two specific initiatives, one focused on climate change and the ocean and the other focused on ocean observing, might provide some potential opportunities to support marine microbiome STI across the Atlantic (https://allatlanticocean.org/news/conclusions-from-the-2023-all-atlantic-ocean-research-and-innovation-alliance-forum/). The Biotecmar (https://allatlanticocean.org/wp-content/uploads/2023/07/Marine-Biotechnology-Initiative.pdf) and the Atlantic Ocean Research Alliance (AORA) Marine Microbiome Working Group (https://www.marinemicrobiome.org/) were launched, respectively, with a focus on biotechnology and marine microbiomes. Ongoing data collection for harnessing marine microbiomes is underway in several initiatives across the Atlantic (https://oceandecade.org/actions/observing-and-promoting-atlantic-microbiomes/; https://ocean.si.edu/ocean-life/microbes/marine-microbes; https://www.macumbaproject.eu/; https://jpi-oceans.eu/en/pharmasea; https://oceandecade.org/actions/mission-ocean-microbiomes-tara-microbiomes/; https://triatlas.w.uib.no/; https://www.bluetools-project.eu/; https://marblesproject.eu/). These projects will bring new insights into the use of marine microbiomes in bioeconomy. The European Union (EU)‐lead coordinated microbiome research projects, such as Astral (https://www.astral-project.eu/) and Innoaqua (https://innoaquaproject.eu/), have provided new insights regarding ocean processes, food security, aquaculture, and how a healthy ocean is necessary for a sustainable blue bioeconomy^8^, ^13^.

In addition, the strategic use of shared infrastructure has led to improved insights. For instance, the Geotraces (https://www.geotraces.org/) and the EU‐funded AtlantECO (https://www.atlanteco.eu) projects have relied on the development of shared protocols to leverage expensive infrastructure across underexplored regions in the Atlantic. These efforts have led to increased research in different countries including participation by researchers in the Global South. However, more accessible marine microbiome infrastructure and human capital formation are needed, e.g., through multiuser facilities (https://schmidtocean.org/) and more target programs (http://www.coml.org/). The International Center for Deep Life Investigation at Shanghai Jiao Tong University represents one approach to supporting international collaborations (https://icdli.sjtu.edu.cn/).

Multinational efforts to promote capacity building have involved Brazil, Canada, EU, South Africa, and the United States (e.g., programs Confap‐EU, NSERC/Horizon). The partnership Confap‐EU has engaged in mutual funding of research projects. However, matching funds from all involved countries are required, limiting participation of scientists from many all‐Atlantic partner countries. A key impediment to coordinated research has been the requirement to work “in parallel”, which has led to issues in project codesign and execution of scientific research across the Atlantic^12^. For instance, the projects AQUAUP and BLUEWAYSE, recently implemented under the Sustainable Blue Economy Initiative (https://bluepartnership.eu/news/partnership-decides-first-batch-co-funded-projects), aim to develop microbiome‐based solutions for animal feeding and sustainable aquaculture. However, these European initiatives still lack an all‐Atlantic engagement.

## THE BENEFITS OF ADAPTING THE ALL‐ATLANTIC MARINE MICROBIOME INITIATIVE AND PRIORITIES IN RESEARCH AREAS FOR IMPLEMENTATION

To achieve a sustainable blue bioeconomy, we strongly advocate for the establishment of coordinated international marine microbiome research efforts across the Atlantic Ocean. A focus area on marine microbiomes, similar to what has been accomplished on climate change and ocean observing (https://allatlanticocean.org/news/conclusions-from-the-2023-all-atlantic-ocean-research-and-innovation-alliance-forum/), and a strong network including all countries across the Atlantic Ocean will require investments in human capital, infrastructure, and research. We propose the development of bidirectional exchanges to support existing and new collaborative research partnerships. These exchanges may allow the identification of specific needs including shared resources and the development of new technologies. Capacity building programs may, for instance, focus on the development of artificial intelligence, new equipment and tools, and research focused on poorly characterized regions including the deep sea and corals reefs^14^, ^15^. Artificial intelligence applied on open science resources may allow relevant discoveries of novel microbial genes^16^. Existing programs across the Atlantic (e.g., FULBRIGHT and PROBRAL‐CAPES) could prioritize marine microbiome.

Marine microbiome research requires equipment that may be complex to operate and expensive for researchers in many countries. Developing agreements to facilitate sharing of infrastructure and its associated costs may help to address these challenges. Strong efforts are needed to reduce geographical sampling biases and increase temporal scales in microbiome research. An important aspect of increasing geographical coverage of data is to ensure that the required resources including technology, infrastructure, and data are accessible through partnerships^17^.

Overall, the All‐Atlantic Declaration presents a potential opportunity to go forward. It may underpin the development of strong STI projects, which may support international and interdisciplinary marine microbiome research to benefit the blue bioeconomy for all countries in the Atlantic. However, broad engagement needs to be promoted. The needed (inter)national financial mechanisms should be based on cocreation and codevelopment. Meanwhile, new agreements that would enable truly collaborative research across the Atlantic would facilitate resource sharing, make technologies more accessible, and permit sharing of infrastructure such as equipment or ships. Furthermore, research in the South has been seriously hampered by lack of funding. Cocreation and codevelopment entails participation not only in research funding but also in science and innovation from all nations, at all levels of government. Specific funding programs that close these gaps are required, and south governments need to invest in international bilaterial and multilateral programs. Funding needs to be targeted to support research in South and Central America and Africa if an embracing network inclusive of developing countries such as Brazil, Argentina, Colombia, the Dominican Republic, Ghana, Morocco, and South Africa is to be built. Tangible long‐term commitments to fund marine microbiome projects are needed particularly in developing countries.
